# Exercise-related immune gene signature for hepatocellular carcinoma: machine learning and multi-omics analysis

**DOI:** 10.3389/fimmu.2025.1606711

**Published:** 2025-06-20

**Authors:** Cheng Pu, Lei Pu, Xiaoyan Zhang, Qian He, Jiacheng Zhou, Jianyue Li

**Affiliations:** ^1^ School of Martial Arts, Shanghai University of Sport, Shanghai, China; ^2^ The Key Laboratory of Adolescent Health Assessment and Exercise Intervention of the Ministry of Education, East China Normal University, Shanghai, China; ^3^ Department of Preventive Medicine, Suzhou Wujiang District Second People’s Hospital, Jiangsu, China; ^4^ Department of Interventional Medicine, Liyang Hospital of Chinese Medicine, Jiangsu, China; ^5^ Department of Oncology, Jiangsu Provincial Hospital of Integrated Chinese and Western Medicine, Jiangsu, China

**Keywords:** exercise-related immune genes, multi-omics, hepatocellular carcinoma, machine learning, prognosis

## Abstract

**Background:**

Exercise is known to regulate the immune system. However, its prognostic value in hepatocellular carcinoma (HCC) remains largely unknown.

**Objective:**

This study aims to construct a machine learning-based prognostic signature using exercise-related immune genes (EIGs) to predict prognosis in HCC.

**Methods:**

We obtained mRNA-seq and scRNA of HCC from GeneCards, GEO, TCGA and ICGC. EIG were obtained using WGCNA, differential gene expression analysis and CIBERSORT. Univariate COX analysis and 101 combinations of 10 machine learning algorithms were used to construct EIG prognostic signature (EIGPS), and survival analyses were performed. Furthermore, we conducted molecular subtyping, qRT-PCR, biological functions, immune infiltration, drug sensitivity, and single cell analyses on EIGPS.

**Results:**

Using WGCNA, differential gene expression analysis, and CIBERSORT, 59 EIGs were identified, of which 54 were associated with prognosis. EIGPS constructed by 7 EIGs (UPF3B, G6PD, ENO1, FARSB, CYP2C9, DLGAP5, SLC2A1) had the highest average C-index value (0.742), showing good predictive performance independent of clinical features. qRT-PCR results showed that CYP2C9 was lowly expressed in HCC cells, while all other genes were highly expressed. 7 EIGs were divided into two subtypes, with C2 exhibiting better anti-tumor immunity. Immunological biological differences between high- and low-risk groups based on EIGPS involved immune responses. EIGPS was mainly expressed in macrophages. The high-risk group had higher macrophage abundance and immune escape ability, as well as greater sensitivity to Afatinib and Alpelisib.

**Conclusions:**

We identified key EIGs and constructed an EIGPS that can effectively predict the prognosis of HCC, which offers avenues for better personalized treatments.

## Introduction

Hepatocellular carcinoma (HCC), the most common primary liver cancer, had a global incidence of about 900,000 and caused approximately 830,000 deaths in 2020. The incidence of HCC is growing rapidly, with an estimated incidence of over 1 million cases by 2025 (1, 2). Surgery combined with radiotherapy and chemotherapy remains the mainstay of clinical treatment, with drug being the primary therapy for advanced patients to ensure quality of life. Unfortunately, 5-year survival rate ranges from 13% to 36% from early to late stages (3). Therefore, developing new biomarkers and prognostic model has great clinical implications.

Exercise-induced immunoregulatory changes are associated with cancer progression. Our earlier studies reported changes in multiple immune-related pathways and gene expression after exercise. In animal models, voluntary exercise induced immune cell infiltration into tumor tissue and reduced tumor incidence and growth by 60%, implying that exercise is more than preventive, it may also be therapeutic (4). Immune cells from blood collected after exercise could be utilized as adoptive cell therapy for cancer (5). Additionally, while chemoradiotherapy can stimulate the immune system by increasing tumor antigenicity and altering adjuvants, it may cause some side effects, whereas exercise-induced immune changes seem unlikely to have adverse effects (6). Taken above, the value of exercise-related immune genes (EIGs) in cancer warrants further exploration; however, their value in HCC has rarely been reported, and their prognostic value remains unclear.

In this study, we aimed to identify EIGs associated with HCC prognosis through WGCNA, immune infiltration, and univariate COX analysis. Subsequently, we constructed a prognostic model using machine learning methods and evaluated its performance. Based on the prognostic model, we further performed functional and pathway analysis, single cell analysis, nomogram construction, and explored its immune functions, tumor mutational burden, and drug sensitivity.

## Method

### Data collection and processing

**Figure 1:**
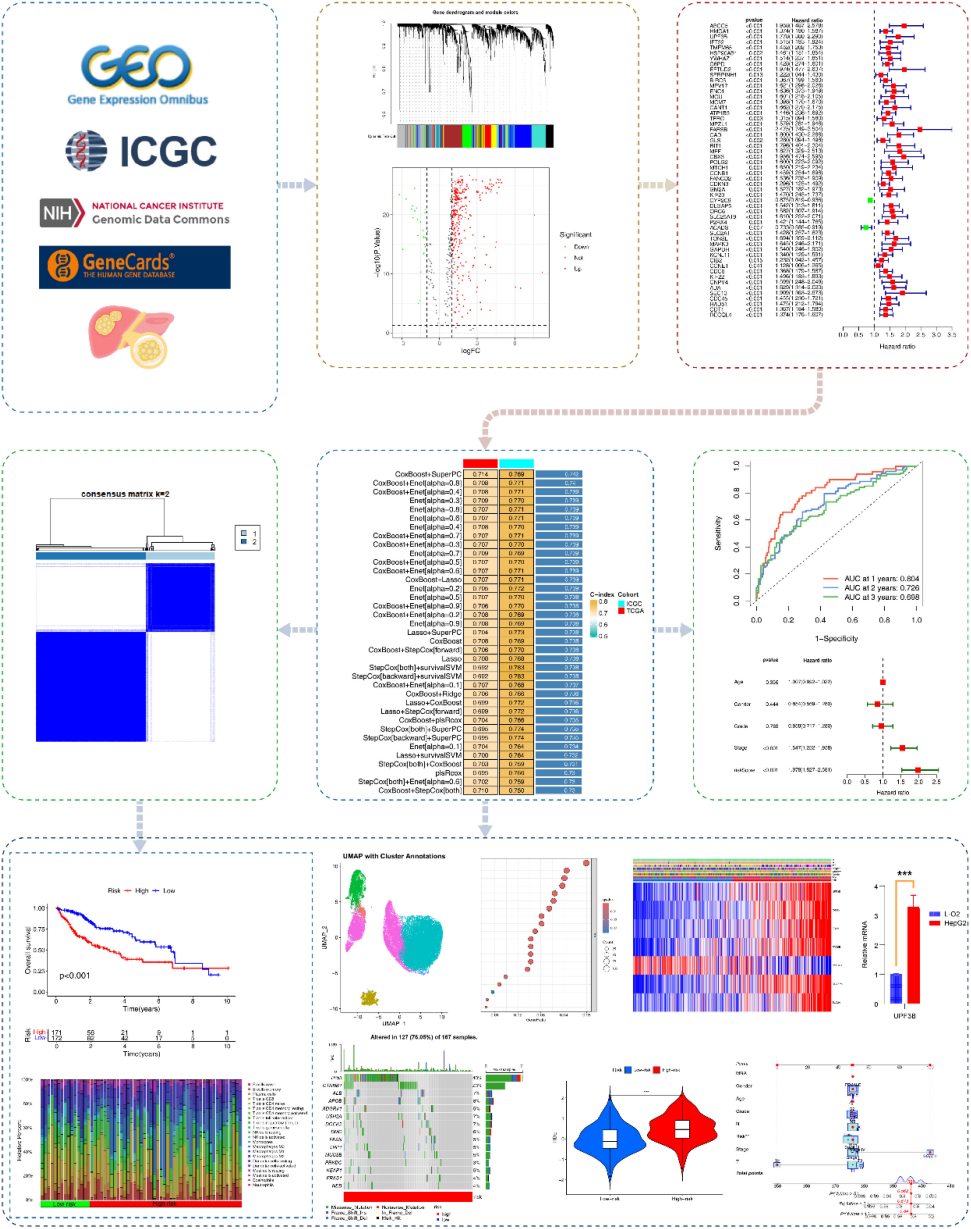
Research flowchart. Based on GeneCards, ICGC, TCGA, we acquired exercise-related genes in HCC patients. Subsequently, EIGs closely associated with HCC were identified using WGCNA, differential gene analysis, immune infiltration analysis, and univariate COX analysis. Multiple machine learning algorithms were applied to construct EIGPS, which was further validated in the validation set. We also performed molecular subtyping, qRT-PCR, survival analysis, clinical feature analysis, tumor mutational burden, immune infiltration, immunotherapy analysis, single-cell analysis, and construction of nomograms.

**Figure 1** presents the research flowchart**. Supplementary Table S1** was the List of Abbreviations. RNA-seq, mutation data, and clinical information were obtained from The Cancer Genome Atlas (TCGA) (424 HCC cases; https://portal.gdc.cancer.gov/v1) as the training set. We obtained 6908 exercise-related genes from GeneCards (search term: exercise; score: 1; www.genecards.org; **Supplementary Table S2**). HCC RNA-seq and clinical information obtained from the International Cancer Genome Consortium (ICGC) were used as the external validation set (233 HCC cases; https://docs.icgc-argo.org/docs/data-access/icgc-25k-data).

### Weighted gene co-expression network analysis

Weighted Gene Co-expression Network Analysis (WGCNA) is a method that constructs scale-free gene co-expression networks to identify functional modules significantly associated with clinical phenotypes and uncover key regulatory genes. In our study, WGCNA was performed to obtain genes most significantly associated with HCC. We obtained gene expression data from TCGA and removed genes with standard deviation of expression level < 0.5 to construct a scale-free co-expression network. The clustering dendrogram was cut at a height of 200 to detect outlier samples. Scale independence and mean connectivity were used to determine the optimal soft threshold, with a scale-free R^2^ of 0.9. The adjacency matrix was converted into a topological overlap matrix (TOM; $\text{T}OM\left(i,j\right)=\frac{\sum_{u}a_{iu}a_{uj}+a_{ij}}{\min\left(k_{i},k_{j}\right)+1-a_{ij}}$ ) and calculated the corresponding dissimilarity (1-TOM). In the TOM formula, the numerator accounts for the direct connection weights and the indirect association strength mediated by all common neighbors, while the denominator acts as a normalization term. To convert TOM into a distance metric, the dissimilarity between two genes is defined as $1-\text{T}OM\left(x,y\right)$ , where $\text{T}OM\left(x,y\right)$ quantifies the similarity between gene *x* and the gene *y* based on their shared topological connectivity patterns. Subsequently, dynamic tree cutting and hierarchical clustering were used to identify modules, with the minimum module size of 50 genes, and the dendrogram was cut at a height of 0.1 to define module clusters.

### Identification of EIGs

We conducted differential gene expression analysis on the module genes obtained from WGCNA analysis with |log2 fold change (FC)| > 1 and FDRq < 0.05. Then, we used CIBERSORT (https://CIBERSORT.stanford.edu/) to assess the infiltration abundance of 22 immune cells in HCC patients. Finally, Spearman analysis was employed to assess the correlation between exercise-related differentially expressed genes (DEGs) and the abundance of 22 immune cells with |r| >0.4.

### Construction of EIGPS

We performed univariate COX analyses to obtain EIGs that were significantly associated with survival in the training set for the construction of EIG-related prognostic signature (EIGPS).

We used 10 machine learning algorithms — LASSO, Ridge, StepCox, CoxBoost, survival support vector machine (survival-SVM), supervised principal components (SuperPC), random survival forest (RSF), generalized boosted regression modeling (GBM), elastic net (Enet), and partial least squares regression for Cox (plsRcox) — to screen variables based on 10-fold cross-validation to construct 101 models. Models containing less than 5 genes were excluded. The optimal model was selected based on the highest C-index. Using the linear combination of the optimal model, we calculated a risk score for each HCC patient, and subsequently divided them into high-risk group (HRG) and low-risk group (LRG). Furthermore, since CoxBoost+SuperPC was the subsequent optimal model, we briefly introduce these two algorithms here. CoxBoost integrates the Cox proportional hazards model with boosting algorithms. Boosting can iteratively optimize regression coefficients to yield a sparse model. SuperPC combines supervised learning with PCA. It first selects features significantly associated with survival outcomes, then applies PCA to reduce dimensionality and extract principal components (PCs) as new features in the subsequent model. This strategy can address high-dimensional collinearity and enhance model robustness.

### Validation of EIGPS

We evaluated the model performance using the training set and the validation set. Principal component analysis (PCA) was used to reduce the dimensionality of gene expression data and project them onto the principal components to identify the distribution of the feature space of HRG and LRG after dimensionality reduction. The K-M survival curve, clinical ROC curve, temporal ROC curve, univariate and multivariate COX analysis, and risk curve were obtained using the “Survival”, “survminer”, and “timeROC” R package. The significance threshold was p < 0.05, and the area under the curve (AUC) of ROC >0.5 was considered significant.

### Correlations between EIGPS and clinical features

We used the chi-square test to assess clinical features in the HRG and LRG, and further conducted survival analysis to examine the associations between clinical features and EIGPS.

### Clustering analysis

Based on the expression of EIGPS genes, we performed clustering analysis to identify molecular subtypes using the “ConsensusClusterPlus” R package with Partitioning Around Medoids (PAM) algorithm and Euclidean distance; 80% of the samples were resampled for 10 repetitions. The optimal number of clusters (k) was determined by cumulative distribution function (CDF) plot and average cluster consensus.

### Functional and pathway enrichment analysis

To further investigate the potential functional and pathway differences between HRG and LRG, we used the “clusterprofiler” R package for GO and KEGG enrichment analysis of DEGs. In addition, gene set variation analysis (GSVA) was used to identify differentially enriched pathways.

### Construction of nomograms

For the sake of individualized prediction, we developed two nomograms for HCC patients. The first nomogram combined risk scores and clinical features, while the second utilized the signature genes. The C-index value was calculated to evaluate the consistency of the predicted values with the observed values.

### Immune-related analyses

We employed CIBERSORT to assess differences in the infiltration abundance of 22 immune cells between HRG and LRG. We used single-sample gene set enrichment analysis (ssGSEA) via “GSVA” R package to assess differences in enrichment levels for 29 immune traits (16 immune cells, 13 immune functions) between HRG and LRG. Additionally, TIMER, CIBERSORT−ABS, QUANTISEQ, MCPCOUNTER, XCELL and EPIC were used as additional immune infiltration algorithms to further validate the results.

Earlier research reported that all tumors could be divided into six immune subtypes, namely, wound healing (C1), IFN-γ dominant (C2), inflammatory (C3), lymphocyte depleted (C4), immunologically quiet (C5), and TGF-β dominant (C6) (7). Therefore, we performed survival analysis based on different subtypes.

To evaluate the association of EIGPS with immunotherapy, we used TISIDB (https://cis.hku.hk/TISIDB/index.php) to acquire immune checkpoint genes and assess the differences between HRG and LRG. The Tumor Immune Dysfunction and Exclusion (TIDE) (http://tide.dfci.harvard.edu/) was used to score the immune evasion ability of HRG and LRG, with higher scores indicating worse response to immunotherapy. The immunophenoscore (IPS) of four immunotherapy regimens for HCC were obtained from the TCIA database (https://tcia.at/home), with higher IPS indicating better response to immunotherapy.

### Tumor mutational burden analysis

Macrophage-related genes were derived from Genecards (**Supplementary Table S3**). TMB was quantified by the number of mutations per megabase. Using the optimal cutoff obtained by “survminer” algorithm, the samples were divided into high TMB group and low TMB group, and were then combined with the risk score for survival analysis.

### Single cell analysis

The scRNA-seq data for HCC were obtained from the China National Gene Bank Nucleotide Sequence Archive (CNSA: CNP0000650; https://db.cngb.org/cnsa) and GEO (GSE162616), including a total of 15 HCC cases.

We used “Seurat” R package to process scRNA-seq data and obtained 34472 genes and 54982 cells. Subsequently, PercentageFeatureSet and FeatureScatter functions were used to calculate the percentage of mitochondrial genes and sequencing depth. We excluded cells with fewer than 20 mitochondria, cells with number of genes < 500 genes or > 10,000, and cells with UMI counts < 500 or > 20,000. The “Harmony” package and PCA were used for batch effect removal and dimension reduction, respectively. “FindNeighbors” and “FindClusters” functions were used for cell clustering and determining the resolution, and Uniform Manifold Approximation and Projection (UMAP) was then used for visualization. Cell types were manually annotated using marker genes. Finally, we performed cell-cell communication analysis using the “CellChat” package, with ligands and receptors pairs from the CellChat database (http://www.cellchat.org/).

### Drug sensitivity analysis

Based on the GDSC database, we used the “oncoppredict” R package to evaluate the sensitivity of HRG and LRG to 198 FDA-approved drugs. Drug sensitivity was evaluated using half maximal inhibitory concentration (IC50), with lower values indicating higher drug sensitivity.

### qRT-PCR and immunohistochemistry

The cell lines (L-O2, HepG2) were purchased from the Institute of Cell Research, Chinese Academy of Sciences. Total RNA was extracted using Trizol (Invitrogen, 1596-026) according to the manufacturer’s protocols. cDNA was synthesized using reverse transcription kit (Fermentas, #K1622). qRT-PCR was performed using SYBR Green kit (Thermo, #K0223). Primers are shown in the **Supplementary Table S8**.

We obtained the immunohistochemical results of the biomarkers in the normal population and HCC patients from the HPA database (https://www.proteinatlas.org/).

### Statistical analysis

All statistical analyses were performed using R software (Version 4.3.2; the R Foundation, St. Louis, MO, USA). Chi-square test was used for categorical data, while t-test or Wilcox test was used for continuous data. Unless otherwise specified, p < 0.05 was the significance threshold.

## Results

### Identification of EIGs associated with HCC prognosis

**Figure 2:**
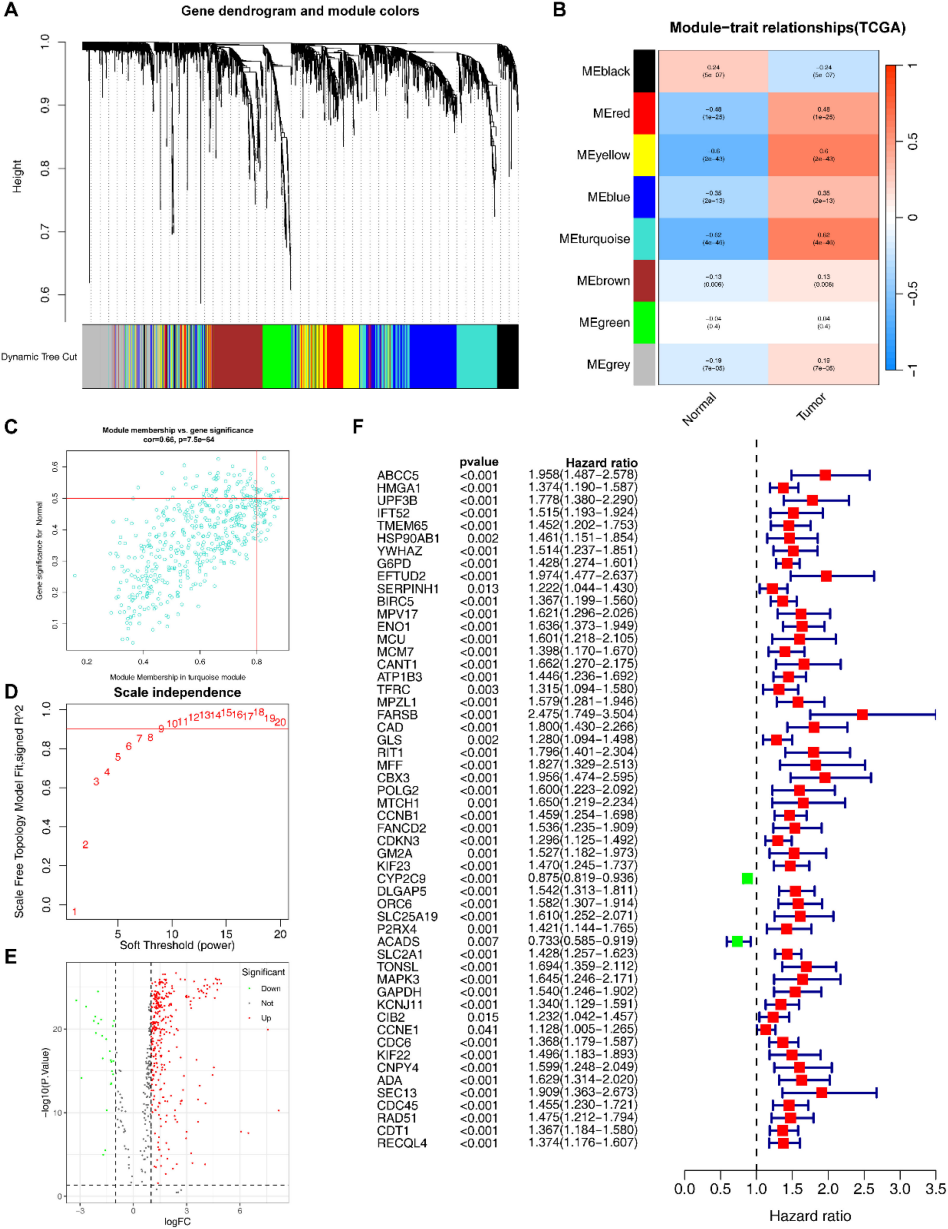
Identification of EIGs. **(A)** Cluster dendrogram of WGCNA analysis of HCC. **(B)** Module-trait heatmap indicating the correlation between modules and HCC. **(C)** Correlation between the gene significance (GS) and module membership (MM) in the turquoise module. **(D)** The selection of soft threshold β. **(E)** Volcano plot of DEGs of HCC. **(F)** Univariate COX analyses associated with survival.

After acquiring exercise-related genes from Genecards, we performed WGCNA using the training set to identify module genes most associated with HCC prognosis. We identified 8 co-expression modules using hierarchical clustering with an optimal power value of 10 (**Figures 2A, B, D**; **Supplementary Table S4**). Among these modules, the turquoise module (500 genes) had the highest correlation with HCC. And the gene significance (GS) and module membership (MM) for the turquoise module showed a significant correlation (r=0.66, P=7.5e-64; **Figure 2C**). We then performed differential gene expression analysis on 500 genes in the turquoise module and identified 343 DEGs (**Figure 2E**; **Supplementary Table S5**).

Subsequently, we assessed the correlation between the identified DEGs and the infiltration abundance of 22 immune cells, and obtained 59 EIGs (**Supplementary Table S6**).

We conducted univariate COX analysis to further identified 54 EIGs that were significantly associated with HCC prognosis (**Figure 2F**; **Supplementary Table S7**).

### Construction and validation of EIGPS through machine learning

**Figure 3:**
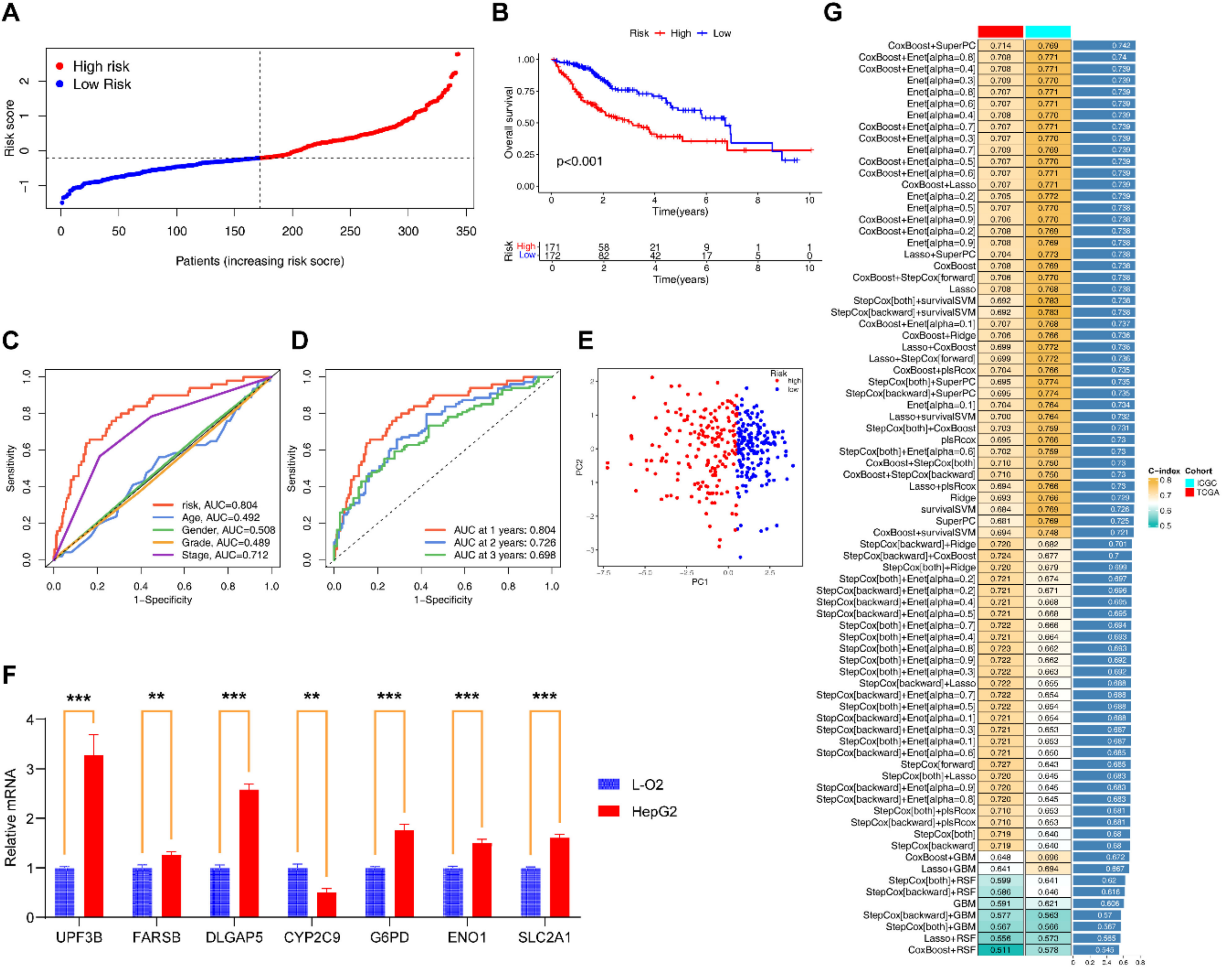
Construction and evaluation of EIGPS. **(A)** Risk curves based on EIGPS. **(B)** K-M survival curves based on EIGPS. **(C)** ROC curves of risk scores and clinical features. **(D)** ROC curves predicting 1-, 3-, and 5-year survival. **(E)** PCA based on EIGPS. **(F)** qRT-PCR of the signature genes (**=P < 0.01; ***=P < 0.001). **(G)** Construction of 101 EIGPS models using integrated machine learning based on 10-fold cross-validation.

Among the 101 models constructed through machine learning, the CoxBoost +SuperPC model had the highest average C-index (0.742; **Figure 3G**), and incorporated 7 EIGs (UPF3B, G6PD, ENO1, FARSB, CYP2C9, DLGAP5, SLC2A1). Low expression of CYP2C9 was associated with poor HCC prognosis, whereas the other genes were the opposite (**Supplementary Figures S1C–I**). qRT-PCR and immunohistochemical results also supported these findings (**Figure 3F**; **Supplementary Table S8**; **Supplementary Figures S2A–G**).

To comprehensively assess the robustness of the constructed EIGPS, we calculated a risk score for each patient and divided all patients into HRG and LRG. Univariate (HR=2.229, 95%CI= 1.773−2.803, P < 0.001; **Supplementary Figure S1B**) and multivariate (HR=1.978, 95%CI= 1.527−2.561, P < 0.001; **Supplementary Figure S1A**) COX regression analyses showed that the risk score was associated with HCC survival independent of other clinical features.

PCA suggested that the risk score could significantly distinguish between HRG and LRG (**Figure 3E**). The K-M curves showed that the survival of LRG was significantly higher than that of HRG (**Figure 3B**). And the mortality gradually increased with the increase of risk scores (**Figure 3A**). The area under the ROC curves and decision curves suggested that the EIGPS had better predictive performance (AUC=0.804) than other clinical features (**Figure 3C**). The time-ROC indicated that EIGPS had good predictive performance in predicting survival at 1 (AUC=0.804), 3 (AUC=0.726), and 5 (AUC=0.696) years (**Figure 3D**).

We further validated the performance of EIGPS in the validation set, and observed that the results were all consistent with those in the training set (**Supplementary Figures S3A–I**).

### Molecular subtyping based on EIGPS

**Figure 4:**
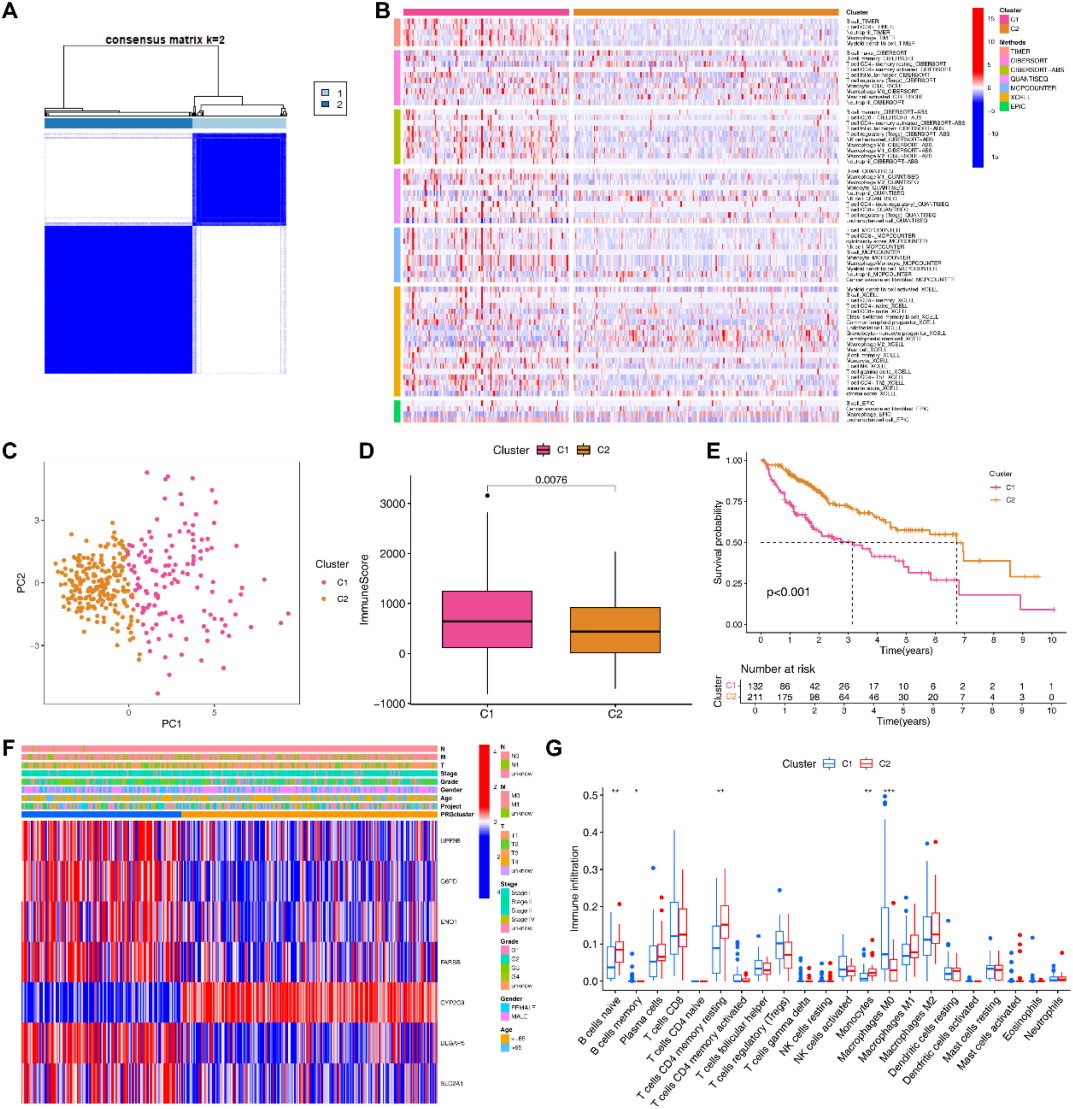
Molecular subtyping based on signature genes. **(A)** Two subtypes of HCC patients based on signature genes. **(B)** 7 immune infiltration algorithms for the two subtypes. **(C)** PCA of the two subtypes. **(D)** Differences in the immune microenvironment between the two subtypes. **(E)** Survival analysis of the two subtypes. **(F)** Heatmap of clinical features and signature gene expression in the two subtypes. **(G)** Differences in the infiltration abundance of 22 immune cells between the two subtypes (*= P < 0.05; **=P < 0.01; ***=P < 0.001).

Based on the expression of EIGPS, we divided HCC samples into 2 subtypes (optimal k=2; **Supplementary Figure S4A**; **Supplementary Figures S4A, B**). PCA showed a clear distinction between the two subtypes (**Figure 4C**). The expression patterns of EIGPS in the two subtypes were similar to risk scores (**Figure 4F**). The C2 subtype corresponded mainly to the LRG and had higher survival rates than C1 (**Figure 4E**). As for tumor immune microenvironment, C2 had significantly lower immune cell infiltration abundance (**Figure 4D**; **Supplementary Figure S4C**), whereas C1 showed a large number of immune tumor-promoting phenotypes, especially higher macrophage (Mφ) abundance across 7 immune infiltration algorithms (**Figures 4B, G**).

### Correlations between EIGPS and clinical features

We observed significant differences in T-stage, Stage, and Grade between HRG and LRG (**Supplementary Figure S5D**). We combined EIGPS with the significantly different features, and found that LRG combined with any clinical feature in early or late stages had better survival than HRG (**Supplementary Figures S5A–C**).

### Construction of nomograms based on EIGPS

**Figure 5:**
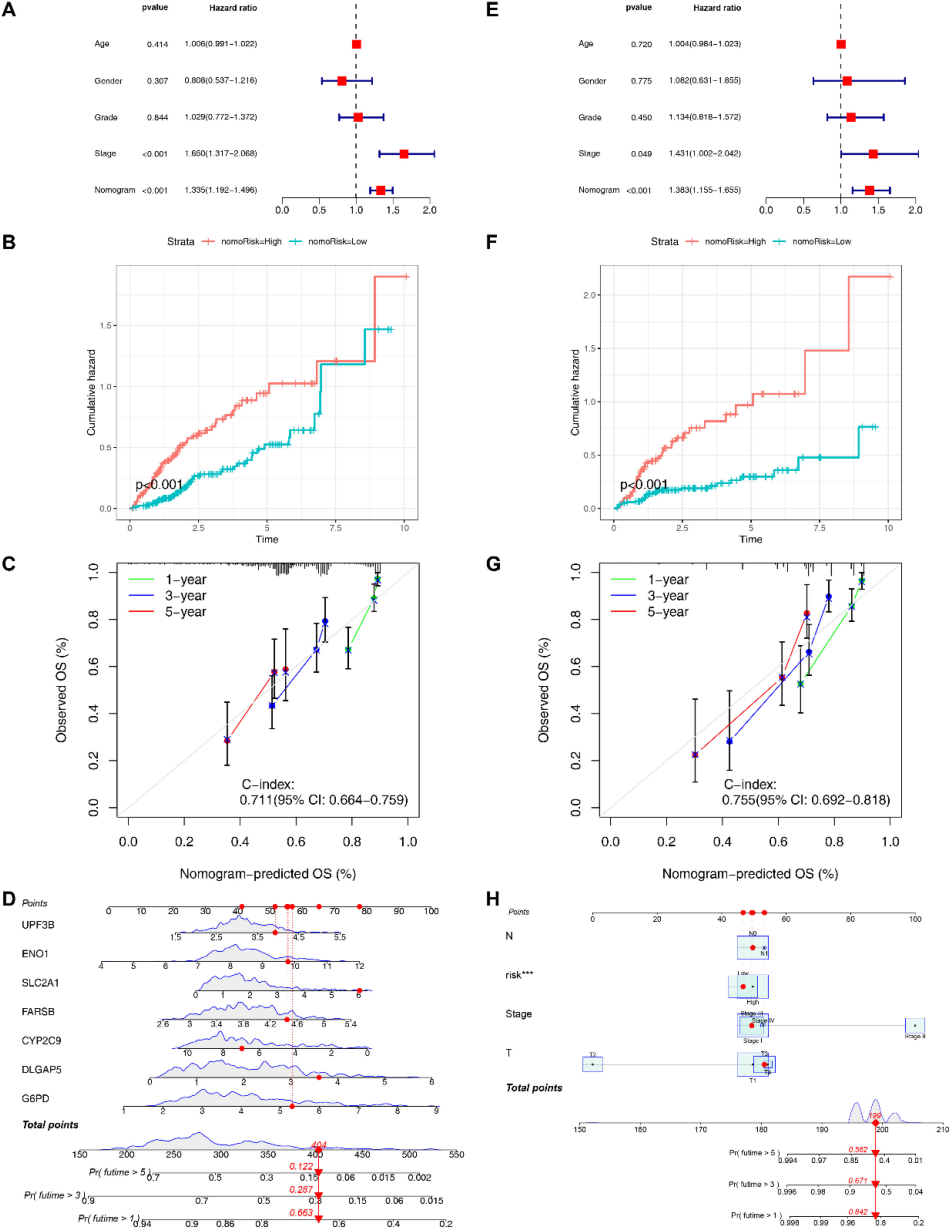
Construction of nomograms. **(A)** Multivariate COX analysis of signature gene nomogram. **(B)** Survival analysis of signature gene nomogram. **(C)** C-index values of signature gene nomogram. **(D)** The signature gene nomogram. **(E)** Multivariate COX analysis of the nomogram based on clinical features and risk scores. **(F)** Survival analysis of the nomogram based on clinical features and risk scores. **(G)** C-index values of the nomogram based on clinical features and risk scores. **(H)** The nomogram based on clinical features and risk scores.

To optimize the clinical application of the risk model, we developed a nomogram based on clinical features and risk scores (**Figure 5H**), which could effectively predict 1-year, 3-year, and 5-year survival (C-index=0.754, 95%CI: 0.703-0.806; **Figure 5G**). We also constructed a nomogram based on EIGPS gene expression (C-index=0.711, 95%CI: 0.664-0.759; **Figures 5C, D**), which may benefit HCC patients who did not have the sequencing of all the 7 signature genes. Both nomograms showed independent predictive ability (**Figures 5A, E**). The mortality gradually increased with the increase of nomogram scores (**Figures 5B, F**).

### Immune infiltration-related analyses based on EIGPS

**Figure 6:**
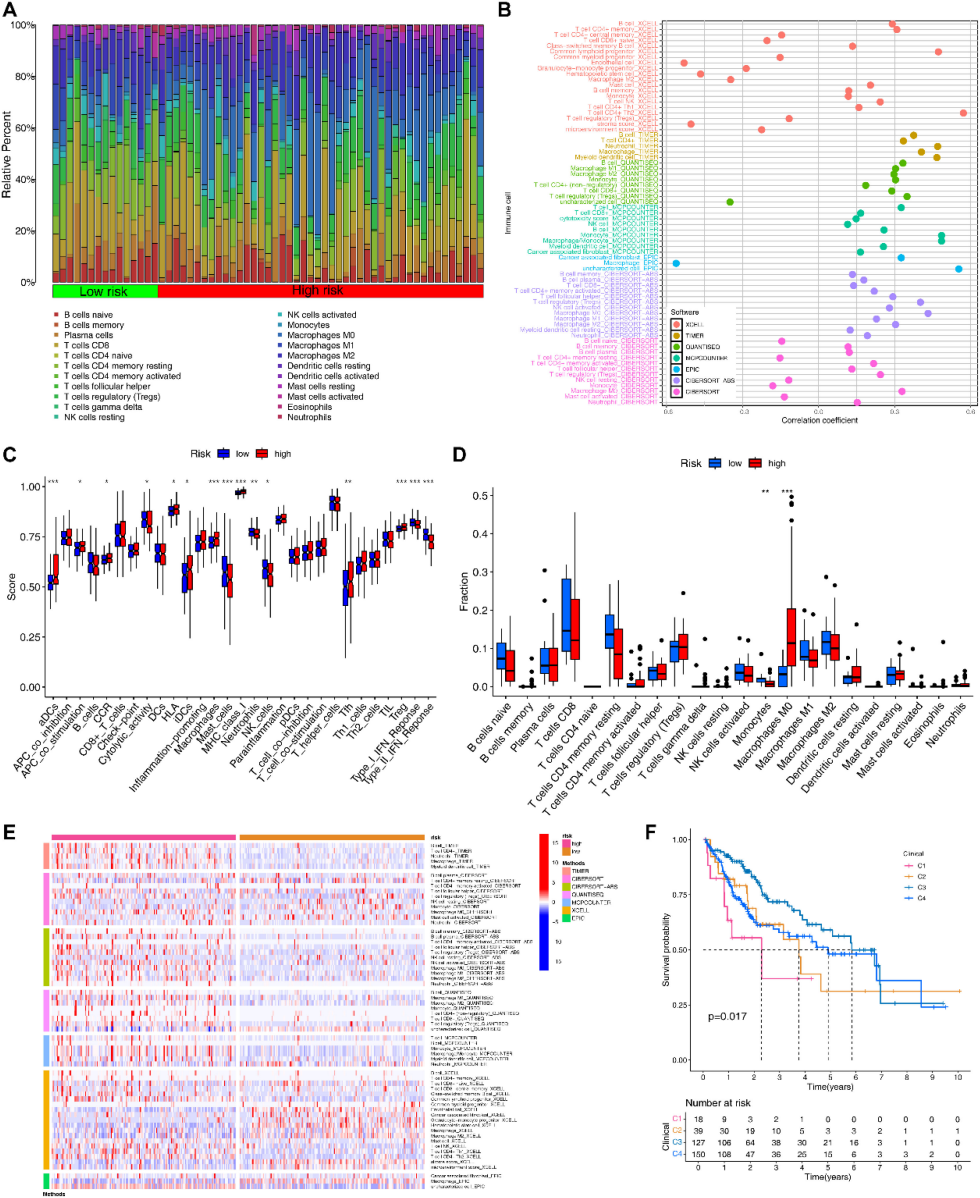
Immune-related analyses based on EIGPS. **(A)** Heatmap of immune infiltration in HRG and LRG. **(B)** Correlation between risk scores and immune infiltration abundance based on 7 immune infiltration algorithms. **(C)** Differences in 16 immune cells and 13 immune functions in HRG and LRG. **(D)** Differences in infiltration abundance of 22 immune cells between HRG and LRG. **(E)** Heatmap of the differences in the immune infiltration abundance between HRG and LRG based on 7 immune infiltration algorithms. **(F)** Survival analysis of immune subtypes.

Based on the immune subtypes identified by the previous study, we found 4 immune subtypes for HCC patients (**Supplementary Figure S6A**), with the C1 and C2 subtypes mainly found in the HRG (**Supplementary Figure S6B**). Patients with C1 and C2 had a significantly worse prognosis compared to other subtypes (**Figure 6F**).

Subsequently, 7 immune infiltration algorithms all suggested a positive correlation between risk scores and macrophage abundance (**Figure 6B**). CIBERSORT results showed that macrophages M0 (Mφ) had significantly higher abundance in HRG than in LRG, while monocytes were the opposite (**Figures 6A, D**). Additionally, the additional 6 immune infiltration algorithms also noted the significant differences in macrophage abundance between HRG and LRG (**Figure 6E**). Similar results were obtained by ssGSEA that the macrophage abundance as well as a number of immune tumor-promoting functions was significantly elevated in HRG (**Figure 6C**).

### Single cell analysis based on EIGPS

**Figure 7:**
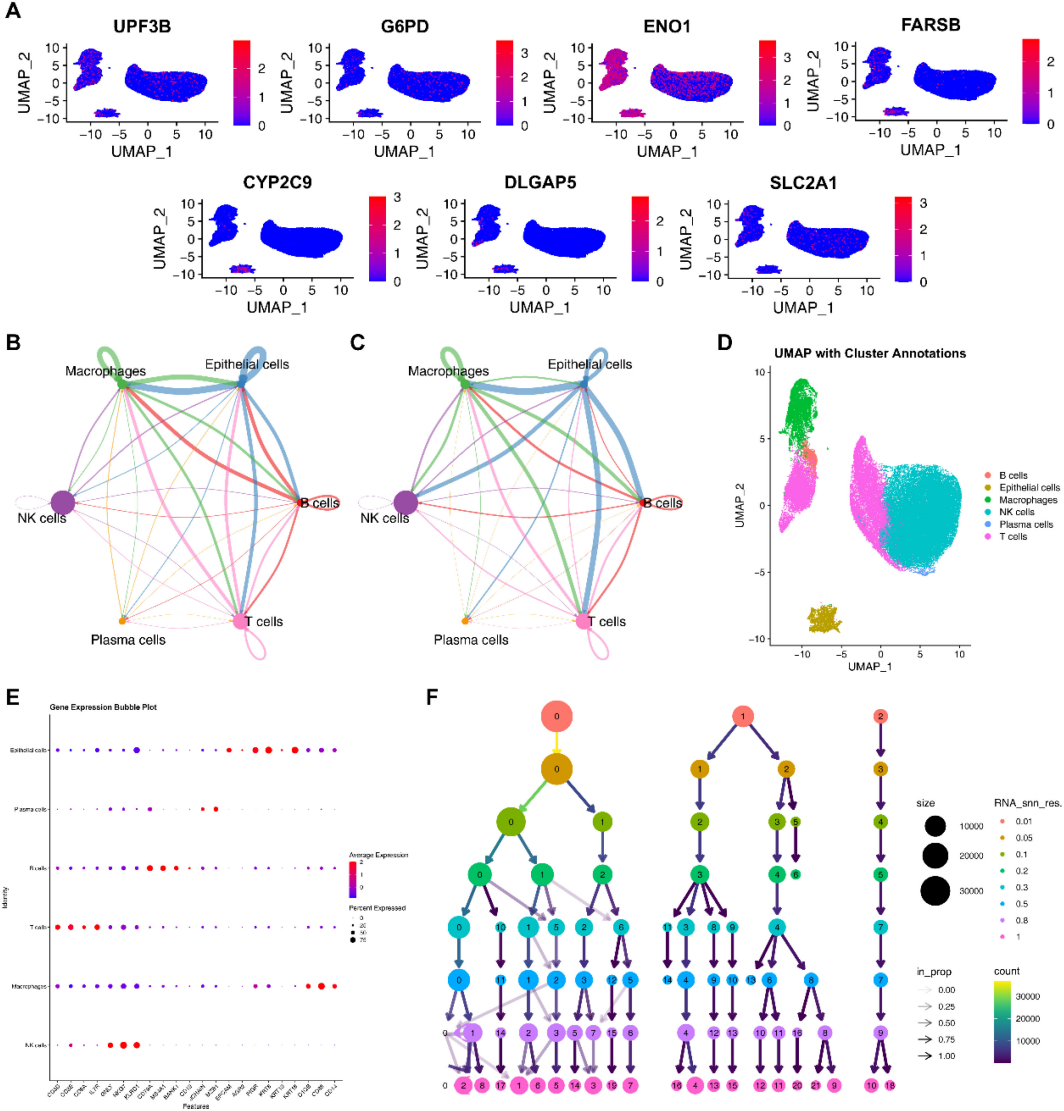
Single cell analysis. **(A)** The expression of signature genes in cells. **(B)** Circle plots of interaction number in cell-cell communication. **(C)** Circle plots of interaction strength in cell-cell communication. **(D)** Annotated cell types using UMAP. **(E)** Marker genes of cell types. **(F)** The selection of clustering resolution.

Based on PCA, the elbow method (selecting the top 10 principal components), and a resolution of 0.8, we obtained 25,664 genes and 50,016 cells, identifying 17 clusters. (**Supplementary Figures S7A, D**; **Figure 7F**; **Supplementary Figures S7B, C**). Subsequently, based on the expression of marker genes, we identified 5 cell types, including B cells, epithelial cells, macrophages, NK cells, plasma cells, and T cells (**Figures 7D, E**). EIGPS were mainly expressed in macrophages (**Figure 7A**). Furthermore, the interaction between macrophages and epithelial cells exhibited the highest interaction number and strength (**Figures 7B, C**). Macrophages mainly functioned as ligand-secreting cells to send signals to epithelial cells, a process potentially mediated by PPIA-BSG ligand-receptor pairs (**Supplementary Figure S8**).

### Immunotherapy-related and drug sensitivity analyses

**Figure 8:**
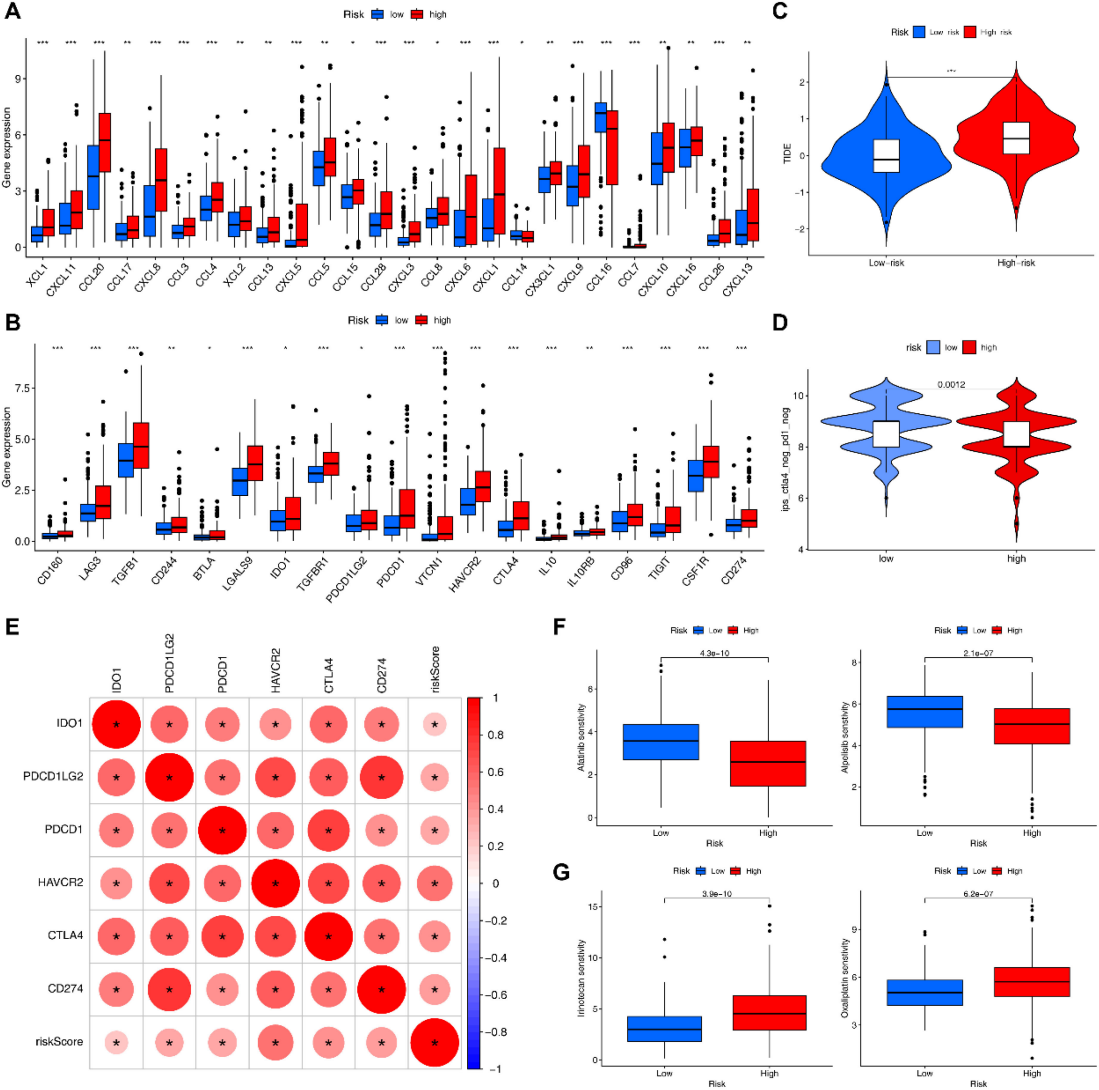
Immunotherapy-related analyses. **(A)** Differential expression of chemokines in HRG and LRG. **(B)** Differential expression of immune checkpoint genes in HRG and LRG. **(C)** TIDE scores in HRG and LRG (***= P < 0.001). **(D)** IPS scores in HRG and LRG. **(E)** Correlation of risk scores with clinically common immune checkpoints. **(F)** Drugs that are more sensitive in the HRG. **(G)** Drugs that are more sensitive in the LRG.

As expected, the expression of immune checkpoint-related genes and chemokine-related genes in the HRG was significantly higher than those in the LRG (**Figures 8A, B**). Among them, we selected immune checkpoint-related genes that were closely associated with clinical treatment, and found that all of them were positively correlated with risk scores (**Figure 8E**). The HRG had a significantly higher TIDE score than LRG, indicating its higher immune evasion ability and poorer response to immunotherapy (**Figure 8C**). The IPS results revealed that LRG had a higher IPS for CTLA4-/PD-1- treatment (**Figure 8D**). Drug sensitivity analysis showed that the HRG was more sensitive to Afatinib and Alpelisib (**Figure 8F**), whereas the LRG was more sensitive to Irinotecan and Oxaliplatin (**Figure 8G**), which provides reference for preferable choices of drugs for different patients.

### Immune-related biological functions and pathways

We further revealed differences in potential biological functions and pathways between the HRG and LRG. GO-BP analysis revealed that DEGs of HRG and LRG were mainly enriched in leukocyte mediated immunity and positive regulation of leukocyte activation (top 2; **Supplementary Table S9**; **Supplementary Figure S9A**). KEGG results showed that the top 2 most involved pathways were cytokine−cytokine receptor interaction and human T−cell leukemia virus 1 infection (**Supplementary Table S10**; **Supplementary Figure S9B**). GSVA enrichment analysis showed that the HRG and LRG had significant differences in complement and coagulation cascades, Fcγ receptor mediated phagocytosis and other immune-related pathways (**Supplementary Figure S9C**).

### Tumor mutational burden analysis based on EIGPS

HCC patients were divided into high TMB and low TMB groups using the best cutoff value (cutoff = 1.316). The K-M curves showed that high TMB group had significantly lower survival rate than low TMB group (**Supplementary Figure S10A**). Furthermore, LRG combined with either low or high TMB had better survival than the HRG (**Supplementary Figure S10B**).

Since EIGPS was mainly expressed in macrophages, we further analyzed 1528 macrophage-related genes, and found that TP53 had highest mutation frequency in the HRG, while the mutation frequency of CTNNB1 was highest in the LRG (**Supplementary Figures S10C, D**).

## Discussion

The incidence and mortality of liver cancer are increasing year by year. Recently, exercise has shown promising therapeutic effects in several cancers. In this study, we used WGCNA to obtain 500 exercise-related genes significantly associated with HCC prognosis. Subsequent differential gene expression analysis, immune infiltration analysis, and univariate COX regression analysis further identified 54 EIGs. 101 combinations of 10 machine learning algorithms were used to construct EIGPS based on 7 EIGs. EIGPS showed better predictive performance than clinical features. Furthermore, EIGPS was closely related to molecular subtyping, immune cell function and level, scRNA, drug sensitivity, and tumor mutational burden.

To the best of our knowledge, this study is the first to construct and apply EIGPS to HCC patients. HCC patients were divided into HRG and LRG based on EIGPS, and HRG had a poorer prognosis. The area under the ROC curve of EIGPS was higher than that of other clinical features, indicating that EIGPS may have better performance and independent predictive ability. The external dataset further validated the results and enhanced the generalizability of the model. Additionally, based on the expression of signature genes, we identified 2 subtypes, of which C2 was mainly found in LRG and had a better prognosis. This also implies that EIGPS can reflect the prognostic differences of different molecular subtypes and provide more precise targeted therapy. Considering the specificity of HCC patients in clinical practice, we constructed nomograms, which contained clinically accessible features that can be used to accurately predict the prognosis of patients.

EIGPS consisted of 6 prognostic risk genes and 1 protective gene. The expression of EIGPS was verified by qRT-PCR, which was consistent with the results of previous findings. UPF3B is significantly highly expressed both *in vivo* and *in vitro*, and is associated with poor prognosis of patients. It may promote HCC progression by binding to PPP2R2C and further activating the PI3K/AKT/mTOR pathway (8). In addition, UPF3B knockdown significantly increases the number of CD45^+^ immune cells and CD45^+^CD3^+^ immune cells (9). G6PD is the rate-limiting enzyme in the pentose phosphate pathway (PPP). Studies have reported that in T cells, inhibition of G6PD activity can block the PPP, deplete NADPH, and reduce the production of inflammatory factors (e.g., IFN-γ and TNF-α) without affecting the proliferation and early activation of T cells. Also, inhibition of G6PD also inhibits respiratory burst in neutrophils by reducing NADPH supply (10). And after G6PD knockdown, the tumor volume and weight *in vivo* were significantly reduced (11). It is well known that ENO1 and SLC2A1 promote HCC progression through glycolysis and mediation of immune escape. SLC2A1 provides substrates for glycolysis by regulating glucose uptake, while ENO1 catalyzes the conversion of 2-phosphoglycerate to phosphoenolpyruvate at the late stage of glycolysis. Their high expression synergistically enhances the Warburg effect (12–14). Furthermore, the overexpression of SLC2A1 seems to be necessary for the drug-resistant target HER2 to promote tumor drug resistance; ENO1 further affects cancer metabolism microenvironment through the crosstalk between glycolytic and phospholipid-synthesizing enzymes, promoting drug resistance and tumor cell proliferation (15, 16). The association between Warburg effect and immunity has been extensively reported. At present, few studies report the association between FARSB and immune infiltration. FARSB is primary responsible for attaching L-phenylalanine to the terminal adenosine of the corresponding tRNA. FARSB knockdown *in vitro* induces G1 phase arrest and impairs the migration ability of HCC cells (17). It may promote HCC progression through the mTORC1 signaling pathway (18). DLGAP5 has been reported to be highly expressed in HCC and associated with poor prognosis. Overexpression of DLGAP5 reduced the infiltration of CD8T cells by inhibiting the TP53 pathway (19). Silencing of DLGAP5 significantly inhibits the growth, migration and colony formation of HCC cells (20). Consistent with the results of earlier studies, low expression of CYP2C9 was found to be associated with better HCC prognosis (21). CYP2C9 is mainly involved in drug absorption, distribution and metabolism, and is closely related to drug resistance in HCC. Its low expression may be caused by the dedifferentiation of cancer cells (22, 23).

The immune features determine the phenotype, prognosis and treatment of HCC. We found that signature genes were highly expressed mainly in Mφ and that Mφ abundance differed significantly between HRG and LRG. In molecular subtyping, C1 and C2 also showed differences in Mφ infiltration abundance. Mφ in HCC are usually classified into two groups: tissue-resident macrophages and monocyte-derived macrophages. Tumor-infiltrating macrophages are negatively correlated with survival rates and exhibit high plasticity. M1 macrophages exert pro-inflammatory and anti-tumor effects, whereas M2 macrophages have the opposite functions. Mφ can activate the NF‐κB signaling pathway by secreting factors such as S100A9 and IL-6, which promotes the stemness of HCC cells and the self-renewal of cancer stem cells (CSCs), thus providing a pro‐tumorigenic niche for early HCC. Meanwhile, HCC cells induce macrophage polarization from M1 to M2 via exosomes and paracrine signals, exacerbating HCC progression (24). In this process, although M1 macrophages can secrete pro-inflammatory factors TNF-α, NO, and IL-12 to activate T cells and NK cells and exert anti-tumor effects, the metabolic reprogramming of tumor cells and macrophages in HCC can reshape tumor microenvironment and promote macrophage polarization to M2 (25). However, it is noteworthy that M1 also seems to have pro-tumor effects, such as promoting HCC cell motility via NF-κB pathway and inducing PD-L1 expression in HCC (26, 27); such pro-tumor effects warrant further investigation. In addition, we found that Mφ may communicate with epithelial cells via PPIA-BSG. PPIA can be secreted from activated Mφ and interact with CD147 (28). CD147 promotes tumor growth, invasion and immune resistance by inducing epithelial-mesenchymal transition and the production and release of matrix metalloproteinases (29). Mutation analysis of macrophage-related genes revealed that TP53 had the highest mutation frequency in the HRG, while CTNNB1 had the highest mutation frequency in the LRG. TP53 mutant cancer cells have been shown to promote macrophage polarization to M2 by secreting factors such as CSF-1, IL-10 and TGF-β (30). Compared with TP53 mutation, CTNNB1 mutation has a better prognosis, but CTNNB1 mutation seems to be a double-edged sword (31). CTNNB1 mutation may induce CCL5 low expression, while CCL5 overexpression has been shown to limit liver regeneration by inhibiting the secretion of hepatocyte growth factor by repair macrophages (32).

GO and KEGG analyses further revealed significant differences between HRG and LRG in leukocyte-mediated immune function and cytokine-cytokine receptor interaction pathway, which are closely linked to each other. Cytokines can regulate migration, activation, differentiation and effector functions of leukocyte subsets by activating downstream pathways such as Jak-STAT; cytokine signals can also be negatively regulated by ubiquitin/proteasome-mediated STAT degradation or by inhibitory proteins, such as PIAS and SOCS, to maintain immune environment homeostasis (33). However, aberrant promoter methylation and modifications in HCC reprogram this regulation and mediate cell proliferation and infiltration of immune pro-tumor phenotypes (34). Furthermore, GSVA found a significantly reduced expression of complement system in the HRG. Almost all leukocyte subsets express receptors for complement activation fragments, yet the complement is double-edged sword. C3b and the anaphylatoxins play a role in activating T cells, and simultaneous binding of C3d fragments on the immunogenic surface to B cells lowers the threshold for B‐cell receptor activation (35). Meanwhile, anaphylatoxins regulate the accumulation and migration of myeloid-derived suppressor cells (MDSCs) and recruit immunosuppressive factors such as TGF-β, IL-10, PDL-1, and CD46, forming an immunosuppressive microenvironment (36, 37). GSVA also suggested a possible over-activation of the immune system in the HRG. For example, the enhancement of Fcγ receptor-mediated phagocytosis and chemokine signaling may cause extensive macrophage infiltration (38). Moreover, activation of T- and B- cell receptor and antigen presentation signaling pathways may be accompanied by T cell depletion, thus creating a niche for immune escape and HCC growth (39, 40). This explains our finding that HRG had significantly higher PD-1/CTLA-4 expression and poorer response to immune checkpoint inhibitors. Finally, we found that the HRG was more sensitive to Afatinib and Alpelisib, whereas the LRG was more sensitive to Irinotecan and Oxaliplatin. These drugs show promising potential for treating HCC independently or in combination with PD-1, CDK4/6 inhibitors (41–44).

To the best of our knowledge, we first identified key EIGs, constructed an HCC prognostic signature based on them, and further revealed the underlying mechanisms. However, our study also has some limitations. Notably, although current evidence supports the involvement of EIGPS in HCC progression, the regulation of EIGPS by exercise may be heterogeneous across different HCC patients, and therefore our results must be interpreted with caution. Additionally, our current results were based on public databases, and validation was restricted to qRT-PCR and immunohistochemistry. Future studies could strengthen these findings through target gene knockdown/knockout *in vitro*/*in vivo* or exercise intervention in HCC models. Furthermore, we must emphasize that our analyses were based on EIGs, but the complexity of exercise determines the specific expression patterns of related genes, resulting in disparities between exercise-responsive and non-responsive populations. The clinical translation rate of exercise therapy in cancer treatment remains low; a major concern is that the effects of exercise-induced acute immune responses on immune phenotypes and functions remain unclear. Although we have identified a significant correlation between EIGPS and macrophages, it remains challenging to predict the direction of macrophage polarization (M1/M2 switch) in actual exercise interventions. Therefore, it is necessary to further validate signature genes using HCC exercise models in future studies.

## Conclusions

Our study identified 7 EIGs to construct an EIGPS with good predictive performance and accuracy, contributing to the prognostic management and treatment of HCC. In future studies, CCK-8, Western blotting, immunofluorescence, flow cytometry, transfection, and transwell assays can be utilize to further validate the invasive ability, immune responses, and underlying mechanisms of signature genes. Furthermore, integrating spatial transcriptomics and single cell analysis will further elucidate the distribution of signature genes and their changes in the microenvironment.

## Data Availability

The original contributions presented in the study are included in the article/**Supplementary Material**. Further inquiries can be directed to the corresponding author.
