# Supplementary material for: Exercise-related immune gene signature for hepatocellular carcinoma: machine learning and multi-omics analysis
Source: Front Immunol. 2025 Jun 20;16:1606711. doi: 10.3389/fimmu.2025.1606711 (PMC12226479; doi:10.3389/fimmu.2025.1606711)
Supplement: Supplementary file 2 [file Image1.pdf]

Supplementary Figures

Exercise-related immune gene signature for hepatocellular carcinoma: machine learning and multi-omics analysis

Cheng Pu[1, #], Lei Pu[2, #, *], Xiaoyan Zhang[2, #], Qian He[3], Jiacheng Zhou[4], Jianyue Li[5]

[1]School of Martial Arts, Shanghai University of Sport, Shanghai 200438, P.R. China

[2]The key Laboratory of Adolescent Health Assessment and Exercise Intervention of the Ministry of Education, East China Normal University, Shanghai 200241, P.R. China

[3]Department of preventive Medicine, Suzhou Wujiang District Second People's Hospital, Jiangsu 215221, P.R. China

[4]Department of Interventional Medicine, Liyang Hospital of Chinese Medicine, Jiangsu, 213300, P.R. China

[5]Department of Oncology, Jiangsu Provincial Hospital of Integrated Chinese and Western Medicine, Jiangsu 210046, P.R. China

*** Corresponding author**

Lei Pu

The key Laboratory of Adolescent Health Assessment and Exercise Intervention of the Ministry of Education, East China Normal University, 500 Dongchuan Road, Shanghai 200241, China.

Email: puleiecnu@163.com

# Co-first authors

Lei Pu, Xiaoyan Zhang and Cheng Pu are co-first authors.

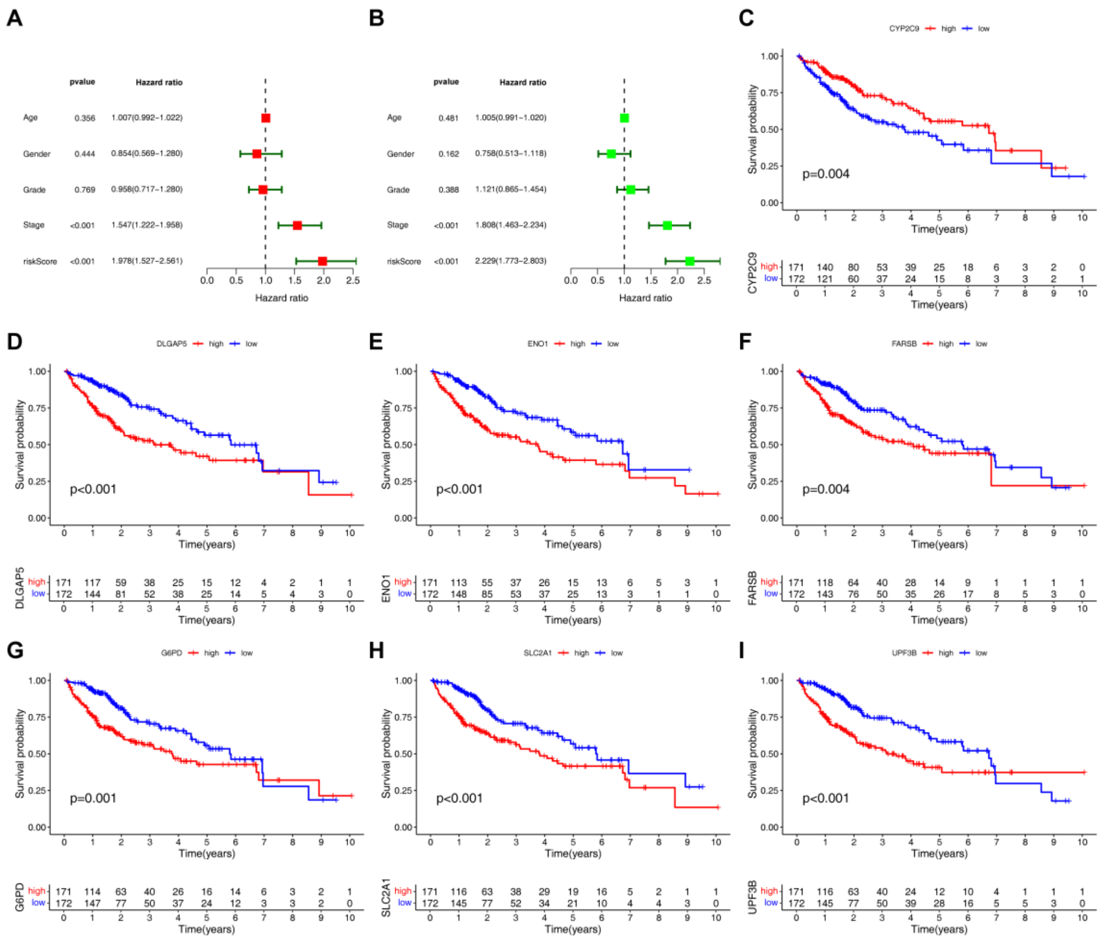

Figure S1. Survival analysis of signature genes, and univariate and multivariate COX analyses of risk scores. (A) Multivariate COX analysis of risk scores. (B) Univariate COX analysis of risk scores. (C-I) Survival analysis of 7 signature genes.

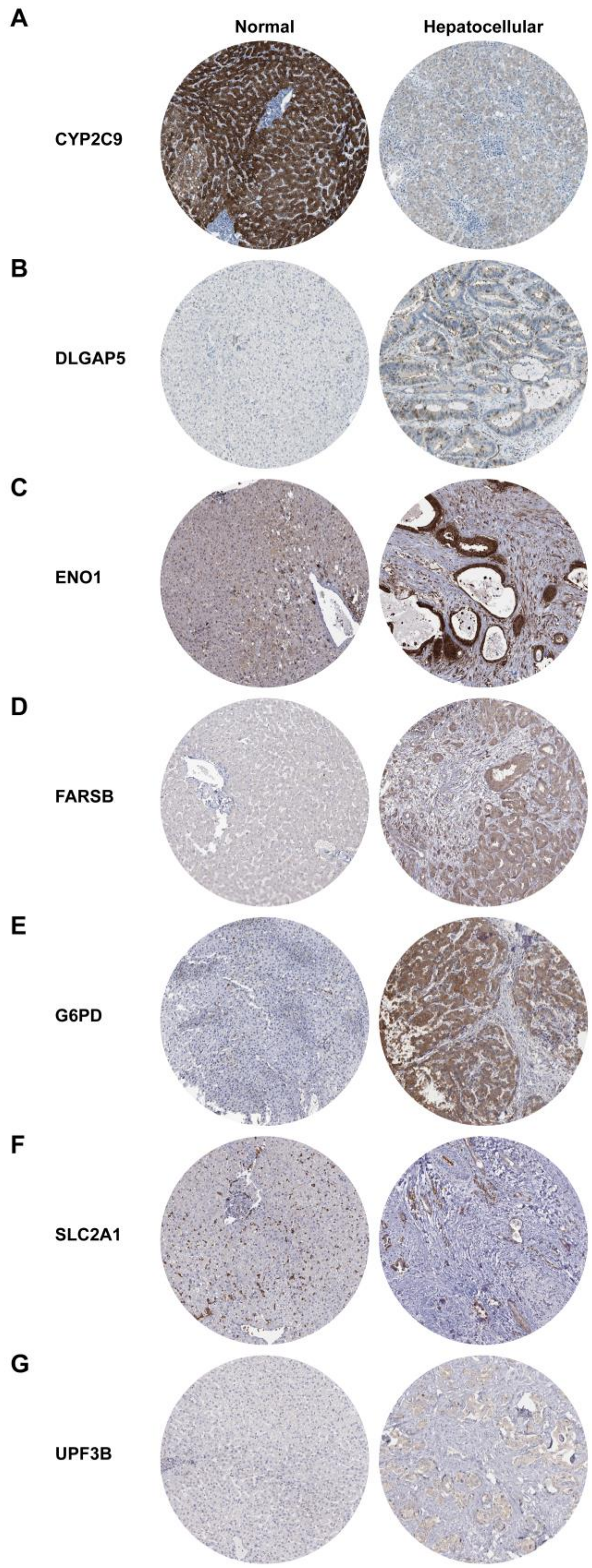

Figure S2. Immunohistochemical analysis obtained by HPA. (A) CYP2C9 was lowly expressed in HCC tissues. (B-G) Six signature genes were highly expressed in HCC tissues.

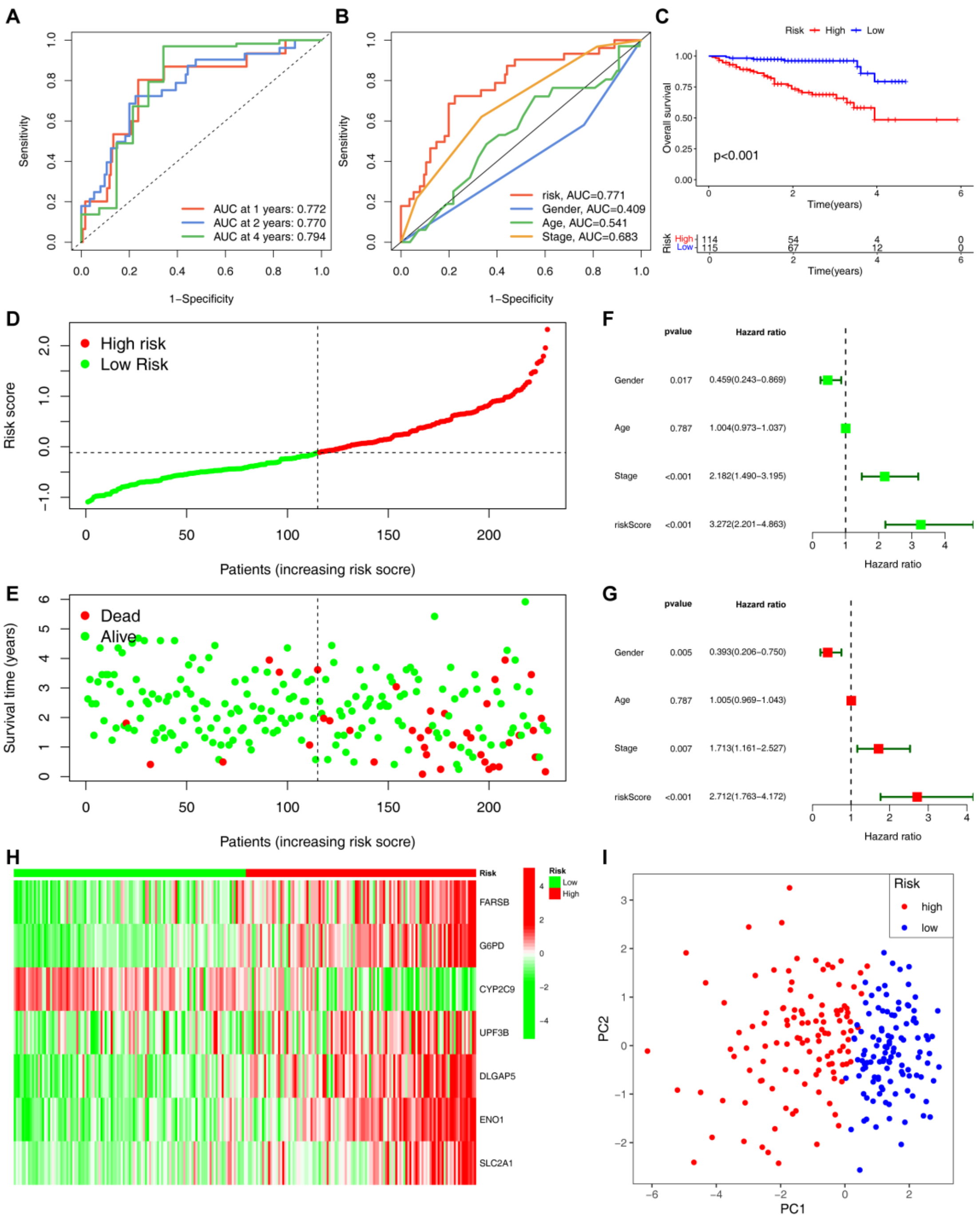

Figure S3. External validation of EIGPS. (A) ROC curves predicting 1-, 3-, and 5-year survival. (B) ROC curves for clinical features and risk scores. (C) K-M survival analysis. (D) Risk score curves. (E) Scatter plot of risk scores. (F) Univariate COX regression analysis of risk scores. (G) Multivariate COX regression analysis of risk scores. (H) Heat map of signature gene expression. (I) PCA based on risk scores.

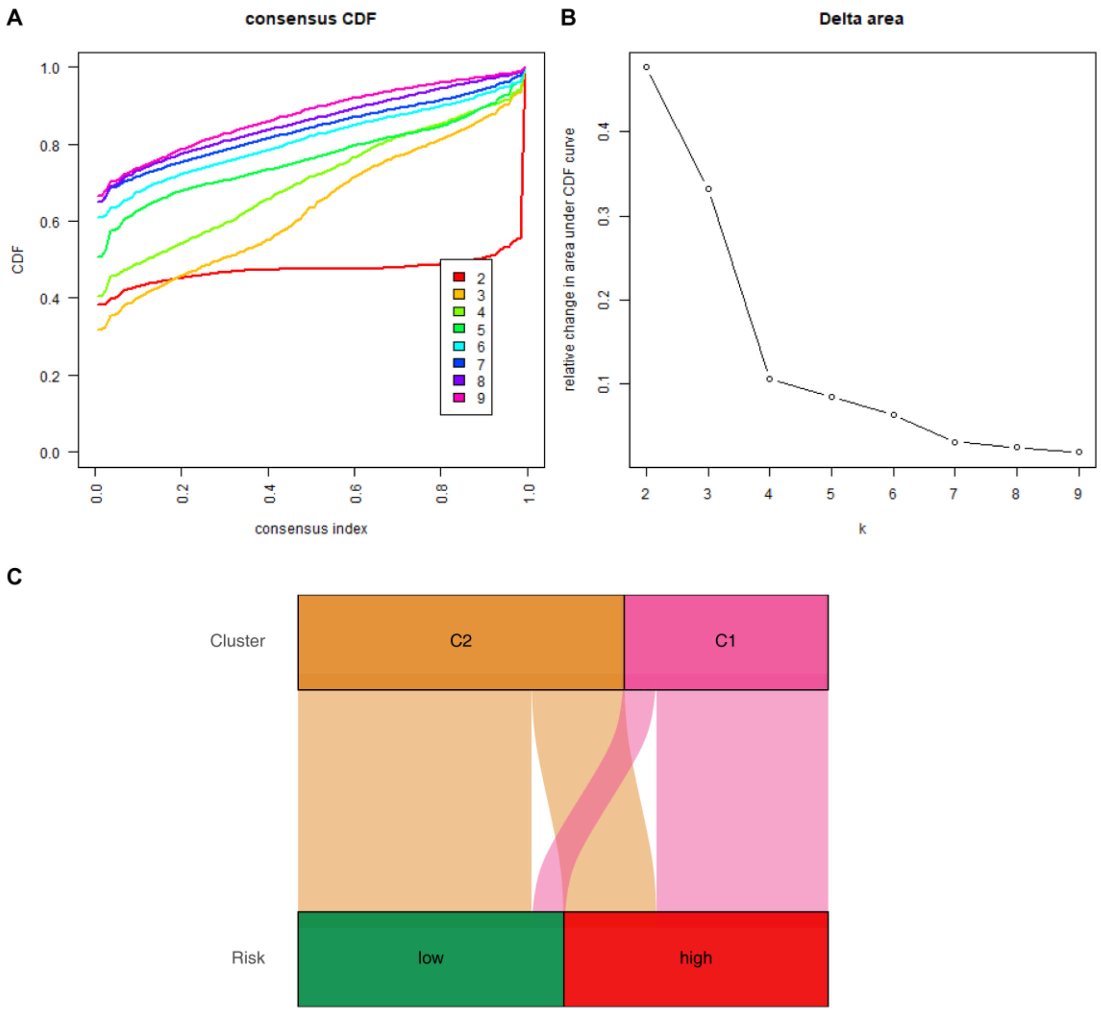

Figure S4. The selection of optimal number of clusters in molecular subtyping. (A) cumulative distribution function (CDF) curves. (B) Relative change in area under CDF curve. (C) Sankey diagram.

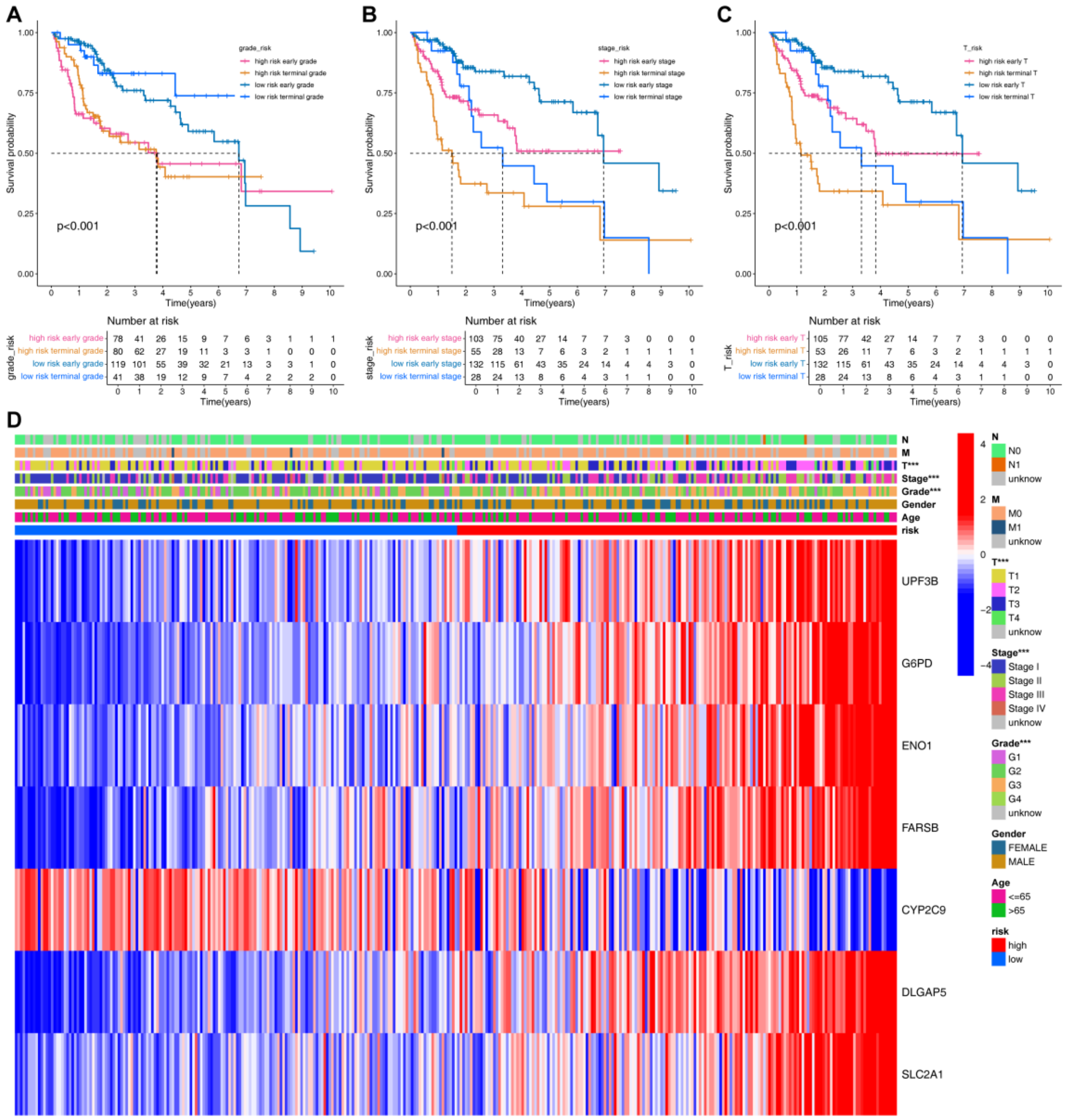

Figure S5. Correlation between clinical features and risk scores. (A) Survival analysis combining risk scores and grade. (B) Survival analysis combining risk scores and stage. (C) Survival analysis combining risk scores and T-stage. (D) Differential analysis of clinical features and heat map of signature gene expression.

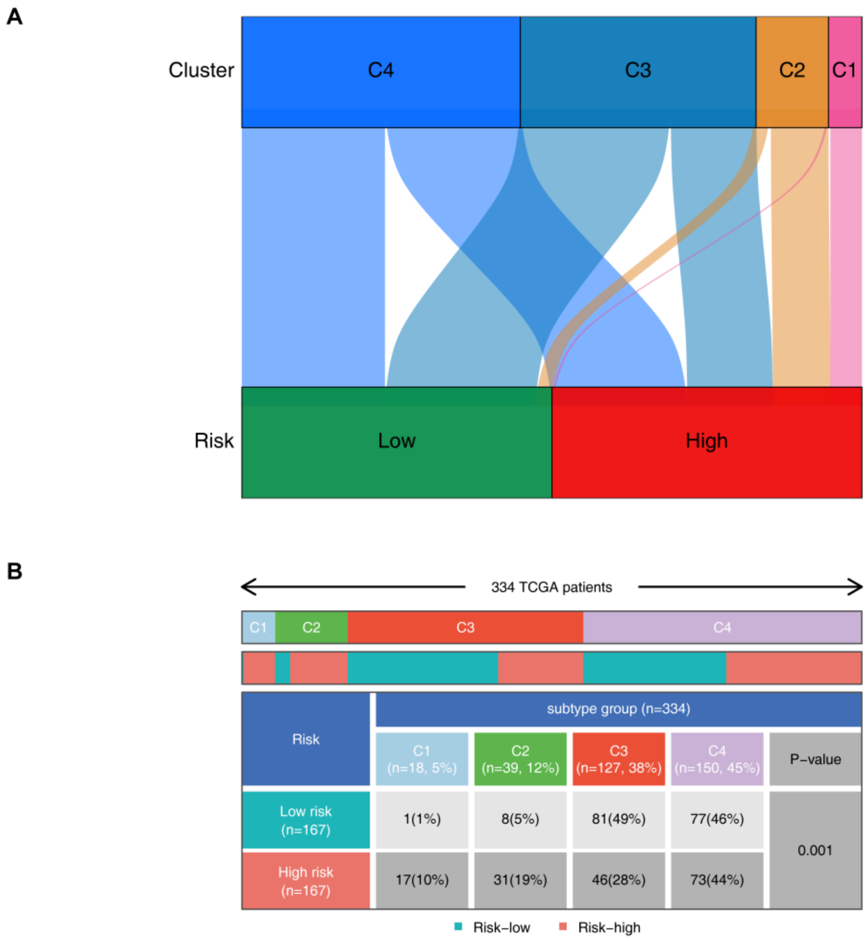

Figure S6. Immune subtyping analysis. (A) Sankey diagram of the subtypes. (B) Distribution differences of subtypes between HRG and LRG.

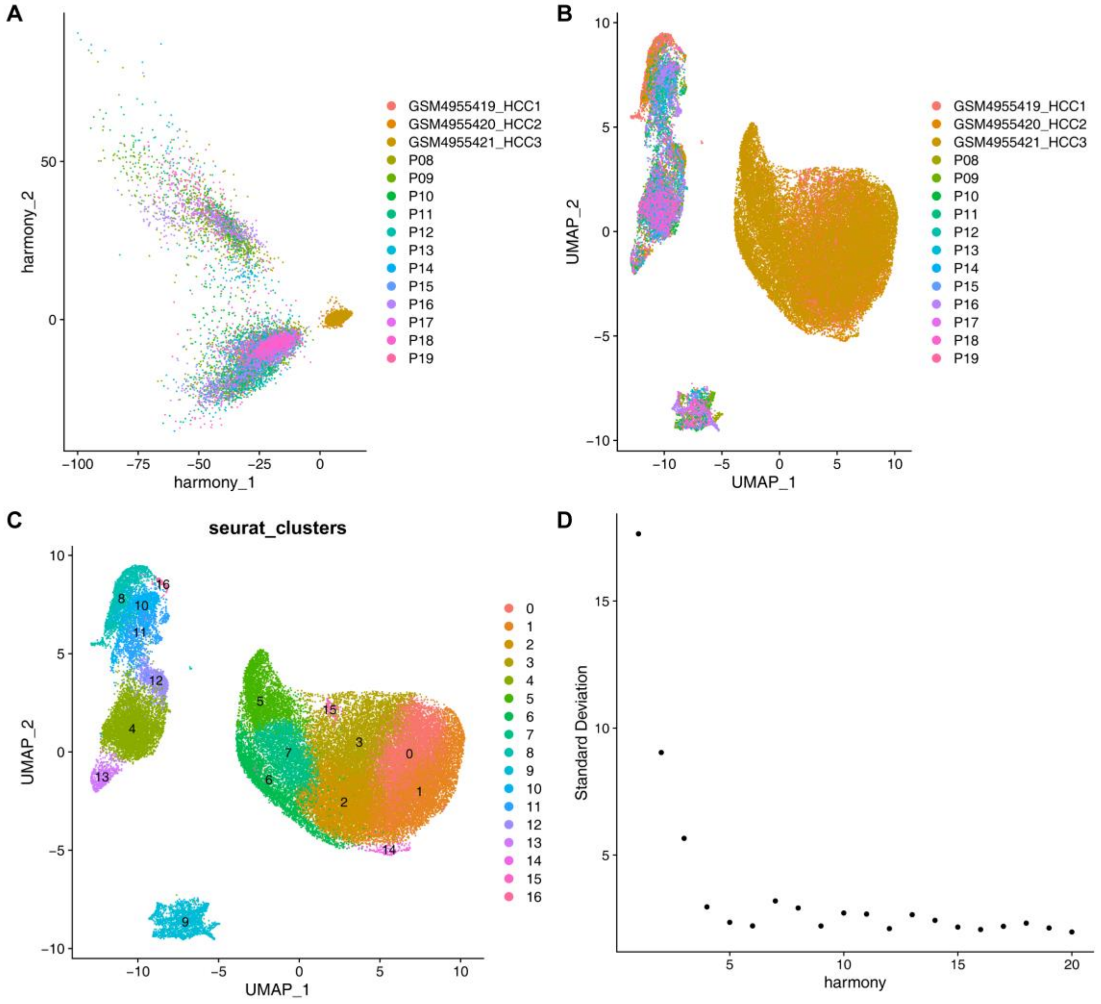

Figure S7. Single cell analysis of HCC. (A) PCA. (B) Clustering analysis. (C) 17 clusters with 0.8 as the best resolution. (D) Elbow plot.

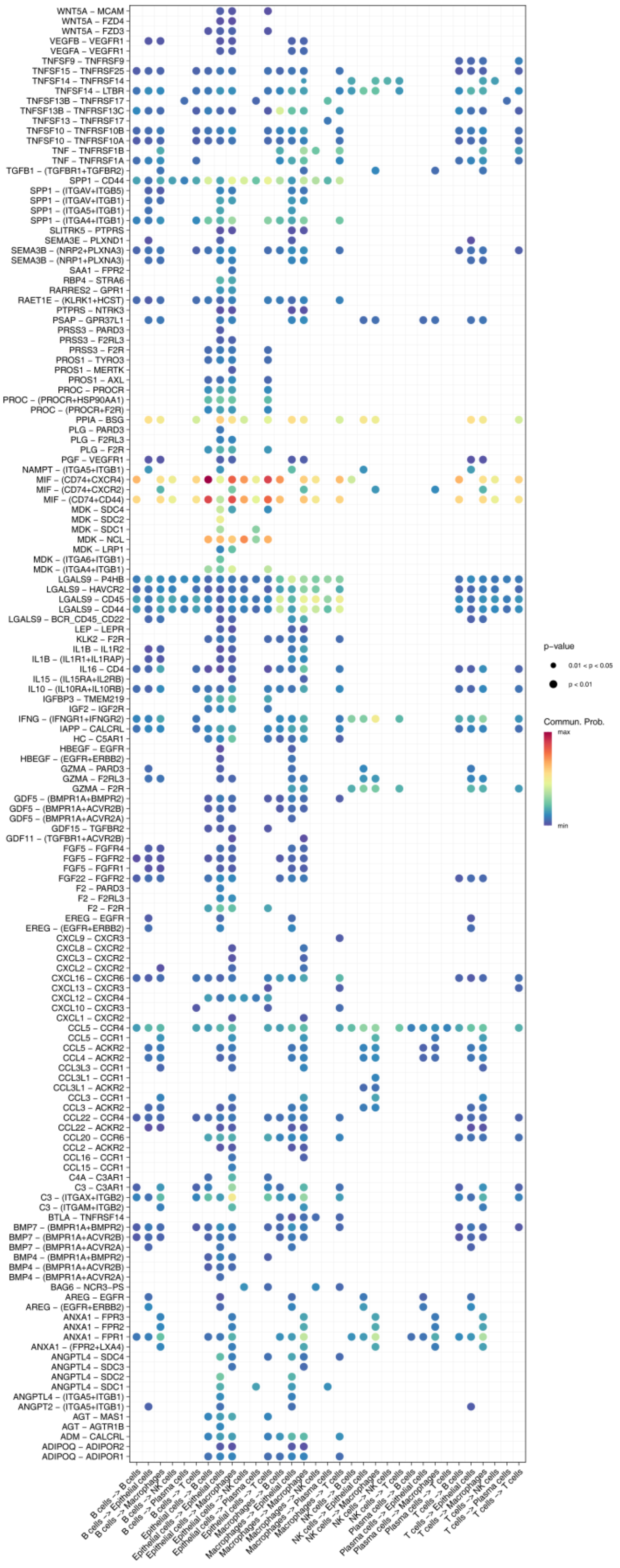

Figure S8. Receptors and ligands for cell-cell communication.

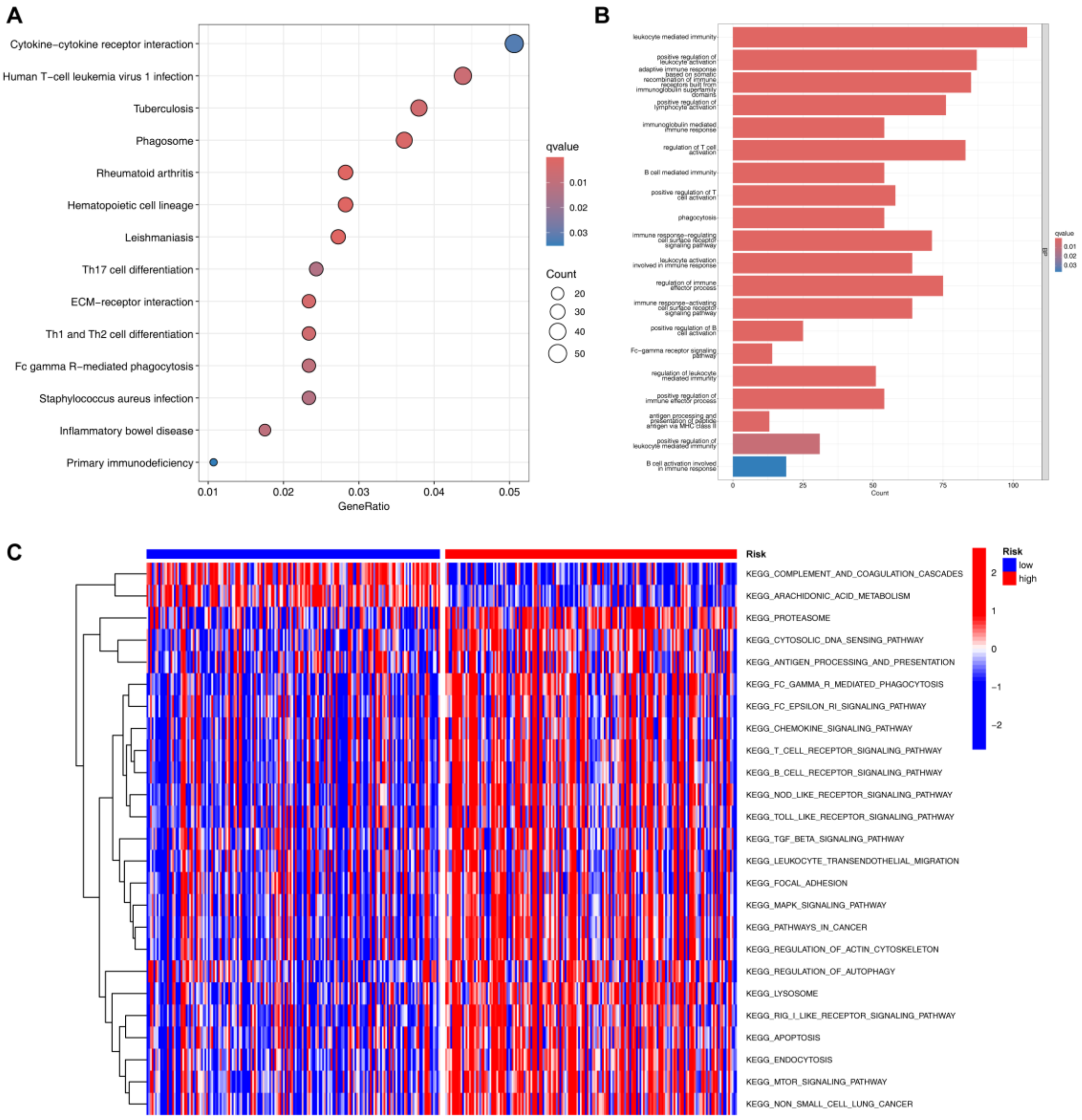

Figure S9. Functional and pathway enrichment analyses of HRG and LRG. (A) GO enrichment analysis. (B) KEGG enrichment analysis. (C) GSVA enrichment analysis.

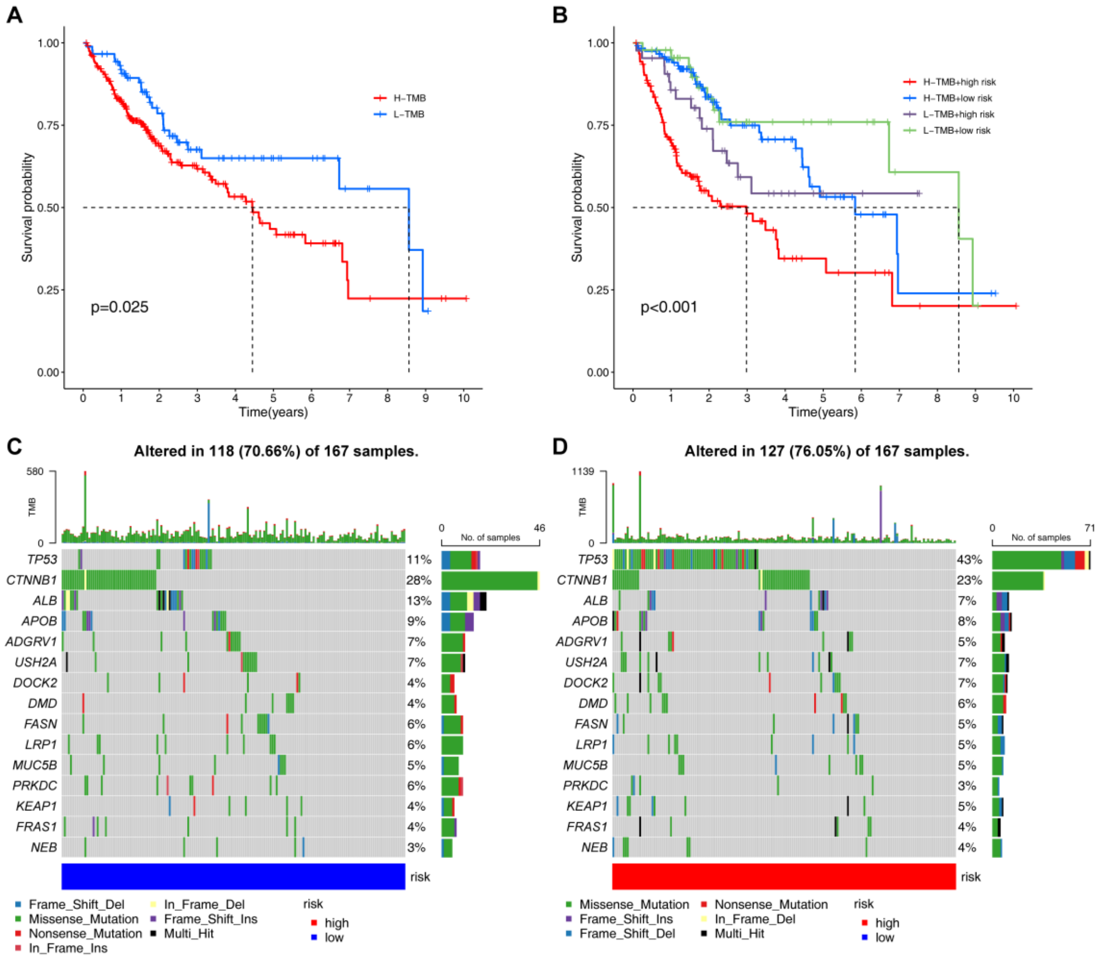

Figure 10. Tumor mutation burden analysis. (A) Survival analysis of high and low TMB groups. (B) Survival analysis combining risk scores and high and low TMB groups. (C) Mutation frequency in the LRG. (D) Mutation frequency in the HRG.
